# A Bone Sample Containing a Bone Graft Substitute Analyzed by Correlating Density Information Obtained by X-ray Micro Tomography with Compositional Information Obtained by Raman Microscopy

**DOI:** 10.3390/ma8073831

**Published:** 2015-06-25

**Authors:** Johann Charwat-Pessler, Maurizio Musso, Alexander Petutschnigg, Karl Entacher, Bernhard Plank, Erik Wernersson, Stefan Tangl, Peter Schuller-Götzburg

**Affiliations:** 1 Department of Forest Products Technology and Management, University of Applied Sciences Salzburg, Markt 136a, Kuchl A-5431, Austria; E-Mails: alexander.petutschnigg@fh-salzburg.ac.at (A.P.); karl.entacher@fh-salzburg.ac.at (K.E.); 2 Department of Materials Science and Physics, University of Salzburg, Hellbrunnerstraße 34, Salzburg A-5020, Austria; E-Mail: maurizio.musso@sbg.ac.at; 3 Department of Engineering and Environmental Sciences, University of Applied Sciences Upper Austria, Stelzhamerstraße23, Wels A-4600, Austria; E-Mail: bernhard.plank@fh-wels.ac.at; 4 Centre for Image Analysis, Swedish University of Agricultural Sciences, Box 337, Uppsala 751 05, Sweden; E-Mail: erik.wernersson@cb.uu.se; 5 Karl Donath Laboratory for Hard Tissue and Biomaterial Research, Department of Oral Surgery, Medical University of Vienna, Sensengasse 2a, Vienna A-1090, Austria; 6 Austrian Cluster for Tissue Regeneration, Donaueschingerstraße 13, Vienna A-1200, Austria; E-Mail: stefan.tangl@meduniwien.ac.at; 7 Department of Prosthetics-, Biomechanic- and Biomaterial Research, Paracelsus Medical University Salzburg, Strubergasse 21, Salzburg A-5020, Austria; E-Mail: peter.schuller-goetzburg@pmu.ac.at

**Keywords:** X-ray micro computed tomography, Raman spectroscopy, bone, Bio-Oss^®^, cluster analysis, image segmentation

## Abstract

The ability of bone graft substitutes to promote new bone formation has been increasingly used in the medical field to repair skeletal defects or to replace missing bone in a broad range of applications in dentistry and orthopedics. A common way to assess such materials is via micro computed tomography (µ-CT), through the density information content provided by the absorption of X-rays. Information on the chemical composition of a material can be obtained via Raman spectroscopy. By investigating a bone sample from miniature pigs containing the bone graft substitute Bio Oss^®^, we pursued the target of assessing to what extent the density information gained by µ-CT imaging matches the chemical information content provided by Raman spectroscopic imaging. Raman images and Raman correlation maps of the investigated sample were used in order to generate a Raman based segmented image by means of an agglomerative, hierarchical cluster analysis. The resulting segments, showing chemically related areas, were subsequently compared with the µ-CT image by means of a one-way ANOVA. We found out that to a certain extent typical gray-level values (and the related histograms) in the µ-CT image can be reliably related to specific segments within the image resulting from the cluster analysis.

## 1. Introduction

Bone is an anisotropic, hierarchically structured material constantly subjected to a renewal process that actively adapts itself to changing mechanical loading conditions throughout its lifetime. The bone microstructure is mainly composed of a collagen matrix, predominantly collagen type I, in which phosphate mineral particles are embedded [1,2]. Water, as the third main component of bone, has a great impact on its mechanical properties since mechanical tests of dry bone exhibit different results compared to those of wet bone. While minerals like carbonated hydroxyapatite provide stiffness, proteins (collagen matrix) yield the required elasticity under wet conditions and thus generate a biological material showing outstanding mechanical properties [3,4].

Bone remodeling (*i.e.*, renewal of bone), which is effected by osteoclasts and osteoblasts, usually increases the mineral content and consequently the mechanical stiffness of bone [2]. A disorder in that cell cycle progression possibly results in skeletal diseases like osteoporosis or periodontosis. Bone grafting is a common way to realize a required bone augmentation via bone transplantation whereat bone graft materials are differentiated into tissues derived from animals referred to as xenograft, tissue taken from another person referred to as allograft, and finally tissue taken from the patient himself referred to as autograft. Autogenous bone (autograft) is still considered as gold standard in medicine, but its usage is restricted due to its limited availability. Consequently, different substitute materials were developed and are still being researched [5,6,7,8].

In medical practice, the quality and the quantity of bone in the jaw is decisive for a successful dental implant. The time of recovery after such a surgical intervention depends on the grade and size of the injury being treated and may vary further from patient to patient.

In order to determine whether healing has sufficiently progressed in order to proceed with implantation, imaging techniques such as X-ray based imaging techniques are standard practice. The information content of X-ray based imaging techniques is restricted to the absorption of X-rays, and consequently provides information on density differences of the matter exposed to them. However, a methodological distinction between original and augmented bone based just on density variations is barely reliable, thus additional imaging information is desirable in order to come to a more sophisticated perception. Specifically, high resolution imaging techniques such as µ-CT have been extensively applied in biomedical research, however, the difficulties in distinguishing autogeneous bone from newly formed bone that are solely based on µ-CT data are mentioned [9,10]. Therefore, many studies used additional imaging techniques in combination with µ-CT in order to draw more precise conclusions [9,11]. Thereby, particular importance was given to histology [12,13,14], as this is considered as gold standard in medicine, however, histology is not a nondestructive method.

Raman spectroscopy is able to provide information on the compositional properties of a sample, [15,16] and has become a major analytical tool to address various biomedical issues [17,18,19,20]. Its use in bone studies ranges from investigations of the compositional properties of materials [20,21,22,23] to biomechanical applications [24,25,26] and the evaluation of bone quality [27,28,29,30].

In this study a porcine bone sample containing the bone graft substitute Bio-Oss^®^ (Geistlich Pharma AG, Wolhusen, Switzerland) was investigated using both, micro computed tomography (µ-CT) and Raman spectroscopy, two nondestructive high resolution imaging techniques capable of providing characteristic density information gained by µ-CT and information on chemical composition obtained by Raman spectroscopy. Firstly, the hypothesis is derived according to the reported benefits of Raman spectroscopy which allows a methodological distinction between autogenous bone and newly formed bone. According to the difficulties addressed in terms of µ-CT, secondly, the hypothesis derives that such a methodological distinction solely based on X-ray absorption represented by gray scale values is not possible. In order to determine whether the hypothesis is to be accepted or rejected these two different information contents were analyzed by means of statistical methods. Moreover, the bone sample used in this study comes from a previous medical study [14] in which newly formed bone could be found. This fact allows the hypothesis to be rejected in case no differentiation could be made between autogenous and newly formed bone by means of Raman spectroscopy.

Raman spectroscopy can be used as an imaging tool [15,16,22,31] (*i.e.*, Raman spectroscopic imaging) as soon as a multitude of spots are measured according to a XY grid within a region of interest (ROI), the result being a so-called Raman map. By focusing on a specific Raman band of interest within the individual Raman spectra of the Raman map, the intensities variations within the map at a given wavenumber position can be displayed in corresponding colors (in our case red meaning high intensity, and blue low intensity), resulting in an image hereafter referred to as Raman image (see e.g., Figure 2 and Figure 3, and the Supplementary Materials). Thus, information on the presence of certain functional groups within the sample can be studied through these Raman images.

The grade of correlation of all the individual Raman spectra within a Raman map with a given reference Raman spectrum can also be coded by colors (in our case red meaning high correlation, and blue low correlation), resulting as well in an image hereafter referred to as Raman correlation map (see e.g., Figure 2 and Figure 3, and the Supplementary Materials).

In this way, the two different information contents are present in image data allowing spatial comparisons. The Raman data set (consisting of the Raman images and Raman correlation maps) was thereby considered in a statistical sense as ground truth, and consequently merged by cluster analysis to a single Raman image consisting of multiple segments (see Figure 6), each representing a chemically different material in order to locate objects or regions of interest [16]. A one-way ANOVA was applied in order to determine how far typical gray level values from the CT image, as a result of X-ray absorption, can be associated with information determined by Raman spectroscopic imaging of relevant chemical structures (see Figure 4 and Figure 5). Although no typical gray level values from the CT image could be found at first unequivocally characterizing the biochemical areas identified by Raman spectroscopic imaging and image segmentation, further data analysis revealed connecting factors (as discussed in conjunction with Figure 6 to Figure 9), suggesting further possible future research efforts in the field of structural and compositional characterization of biomaterials to the benefit of medical and medical related sciences [32,33,34].

## 2. Materials and Methods

The bone sample investigated originates from an adult mini-pig (a native of Minnesota pigs and Vietnamese potbellied pigs), whose sinuses were grafted in a previous medical study [14] with autogenous bone derived cells and bovine bone mineral. This sample was preserved in a 75% alcoholic solution and contained Bio-Oss^®^ as bone graft substitute, and was not embedded in any different material.

**Figure 1:**
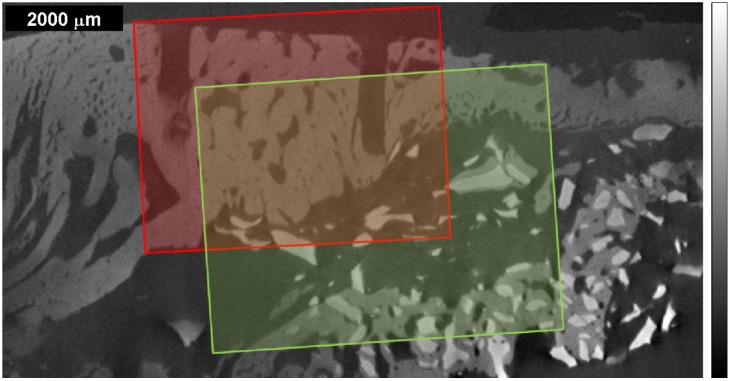
µ-CT image of the bone sample from an adult mini-pig, whose sinuses were grafted in a previous medical study [14] with autogenous bone derived cells and bovine bone mineral. Dark gray corresponds to materials with lower X-ray absorption (e.g., air), while bright gray to materials with higher X-ray absorption (e.g., dense structures). The overlapping regions of interest (ROI) delimit the areas of the Raman maps taken: red area called ROI 1 for the 1st Raman measurement with the wet sample; green area called ROI 2 for the 2nd Raman measurement with the dry sample. The saw kerfs have been used as orientation attributes within the µ-CT image and the Raman maps.

**Figure 2:**
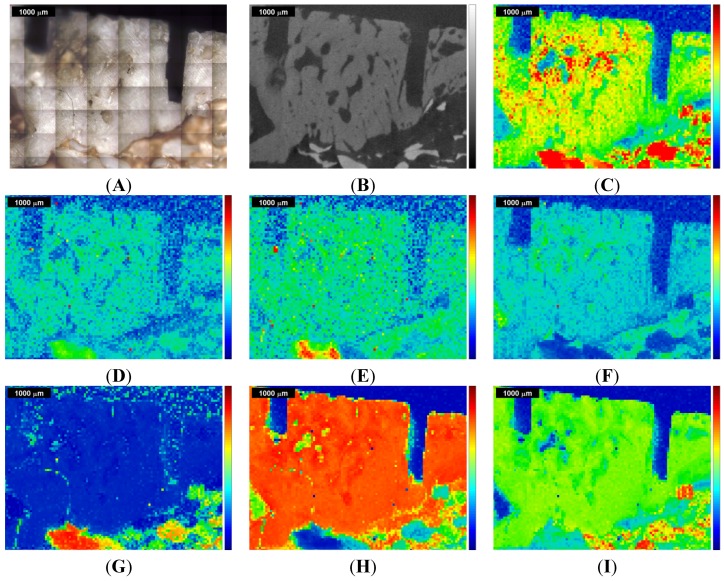
Raman images and correlation maps obtained within ROI 1 from the first measurement of the sinus grafted bone sample (see also Supplementary Materials). Red means high intensity or high correlation, blue means low intensity or low correlation. Upper row: (**A**) microscopic image; (**B**) µ-CT image; (**C**) Raman image centered at the ν_1_PO_4_^3−^ peak at 961 cm^−1^. Mid row: Raman images centered at (**D**) the B-type carbonate substitution peak at 1075 cm^−1^; (**E**) the amide I peak at 1677 cm^−1^; (**F**) the CH stretching peak at 2937 cm^−1^. Lower row: correlation of the individual spectra of the Raman map with the (**G**) Bio-Oss^®^ Raman spectrum; (**H**) natural pork bone Raman spectrum; (**I**) tricalcium phosphate Raman spectrum.

**Figure 3:**
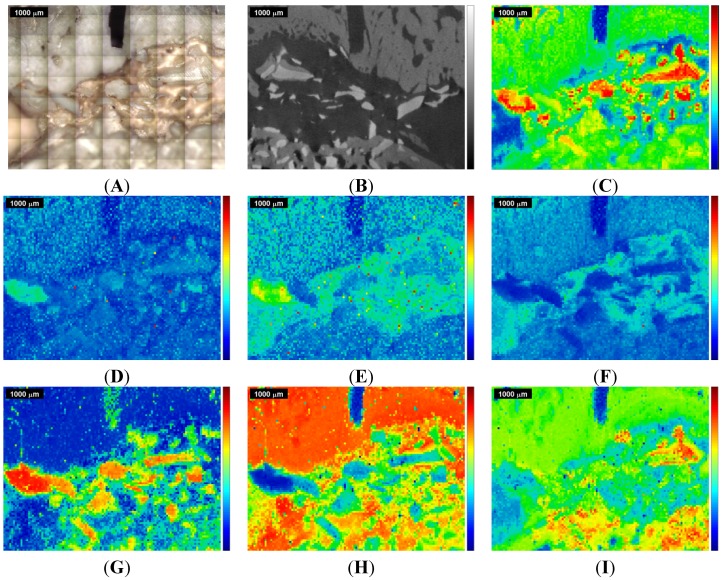
Raman images and correlation maps obtained within ROI 2 from the second measurement of the sinus grafted bone sample (see also Supplementary Materials). Red means high intensity or high correlation, blue means low intensity or low correlation. Upper row: (**A**) microscopic image; (**B**) µ-CT image; (**C**) Raman image centered at the ν_1_PO_4_^3−^ peak at 961 cm^−1^. Mid row: Raman images centered at (**D**) the B-type carbonate substitution peak at 1075 cm^−1^; (**E**) the amide I peak at 1677cm^−1^; (**F**) the CH stretching peak at 2937 cm^−1^. Lower row: correlation of the individual spectra of the Raman map with the (**G**) Bio-Oss^®^ Raman spectrum; (**H**) natural pork bone Raman spectrum; (**I**) tricalcium phosphate Raman spectrum.

The bone sample was examined using both µ-CT and Raman spectroscopic imaging. In order to have the same orientation attribute on the µ-CT images and on the Raman images and Raman correlation maps, the sample was marked with a saw kerf, recognizable in Figure 1, Figure 2 and Figure 3. The µ-CT image was obtained with the help of a GE phoenix|X-ray nanotom 180 NF computer tomograph system at the University of Applied Sciences Upper Austria in Wels (Austria); the sample was scanned in the alcoholic solution with a voxel size of 9.79 μm, the data collection lasting almost 90 min. The tube voltage of the nanofocus tube equipped with a molybdenum target was set at 80 kV. The Nanotom reconstruction software datos|X has been used in order to reconstruct the µ-CT image (see Figure 1) via a back-projection algorithm. The µ-CT values are represented in images with gray scale values ranging from 0 to 65535. In contrast to medical CT scanners, industrial CT do not use calibrated grey values (absorption coefficients) for the bone material. For further image correlation in this work, the 16 bit images were mapped to 8 bit, so that the scale values near 0 (dark gray) correspond to materials with lower X-ray absorption (e.g., air), while scale values near 255 (bright gray) to materials with higher X-ray absorption (e.g., dense bone structures).

The Raman spectroscopic data collection was carried out at the University of Salzburg (Austria) by means of a Thermo Scientific DXR Raman microscope equipped with confocal optics and a 10× objective, a 780 nm laser, and the Thermo Scientific™ OMNIC 8 software for Raman data acquisition and processing. The Raman data set was acquired using a full range grating (50–3300 cm^−1^), and a 50 µm slit entrance at the spectrometer, resulting in an apparatus function with full width at half maximum in the range of 5 cm^−1^ (equivalent to its spectral resolution).

In the sample, the lateral dimensions of the regions of interest (ROIs) covered by Raman mapping had a size of about 4 mm × 5 mm (see Figure 1) with a step size of 50 µm, the Raman maps comprising 7575 spectra for the first measurement (ROI 1, wet sample) and 9890 spectra for the second measurement (ROI 2, dry sample) respectively. In order to have spectra with a reasonable signal to noise ratio (SNR) [35], the acquisition at each single point was set to 8 s and three accumulations for ROI 1 and to 10 s and three accumulations for ROI 2. All recorded spectra were processed by removing fluorescence with an automatic baseline correction [32] provided by the OMNIC 8 software.

As the total measurement times for the Raman maps reached nearly 50 h, the specimen was totally dried up after the first measurement. In order to restrict movements due to shrinkage of the sample, the second measurement was done with the dry sample. Raman measurements within the overlapping ROI (see Figure 1) provided the possibility of comparison between the spectroscopic results of the wet sample and the dry sample. It was found that drying during the first measurement did not impair the results of the second measurement, water being a weak Raman scatterer.

The Raman images were computed by focusing on a specific Raman band of interest inside the individual Raman spectra of the Raman map and show the intensities variations within the map at a given wavenumber position. The Raman bands being of relevance for bone studies are listed in Table 1 and the Raman images of the resulting intensities at a specific Raman band position have been depicted in pseudo RGB colors following the rainbow order, *i.e.*, red revealing a high, green a medium and blue a low intensity at that specific band position. Those images showing a very low contrast have been enhanced by reducing the intensity range [32]. The first three Raman bands listed in Table 1 at 438 cm^−1^, 589 cm^−1^ and 961 cm^−1^, refer to phosphate mineral being an indicator for mineralized organic matrix tissue. The type B carbonate substitution Raman band at 1075 cm^−1^ indicates the presence of carbonated mineral in bone [36]. The following two bands at 1256 cm^−1^ and 1677 cm^−1^ refer to amide being both an indicator for collagenous and non-collagenous (proteins) tissue, as well as for lipids and proteoglycans [23,37], and summarized as matrix in Table 1. The two C-H Raman bands listed in Table 1 represent C-H bending at 1457 cm^−1^ and C-H stretching at 2937 cm^−1^ respectively, and indicate organic moieties denoting collagenous or non-collagenous tissue [23].

**Table 1:** Selected Raman bands of bone [21,23,36] and their intensity ranges for optimum Raman image contrast [32].

Raman Attribution	Peak Position [cm^−1^]	Intensity [cps]	SNR *** ROI 1 ROI 2		Presence * of
ν_2_PO_4_^3−^	438	[0–200]	5.1	5.3	phosphate mineral
ν_4_PO_4_^3−^	589	[0–200]	4.1	5.0	phosphate mineral
ν_1_PO_4_^3−^	961	[0–400]	58	49	phosphate mineral
B-type carbonate sub.	1075	[0–200]	3.7	4.0	carbonate mineral **
Amide III	1256	[0–150]	1.2	2.0	matrix
Amide I	1677	[0–150]	4.8	1.5	matrix
C–H bending	1457	[0–150]	3.0	5.3	matrix
C–H stretching	2937	[0–500]	11	20	matrix

* The phosphate vibrational modes of the hydroxy apatite mainly (PO_4_^3−^) or the carbonate vibrational modes (CO_3_^2−^) describe the presence of mineral; matrix describes the organic moiety (main compound collagen, non-collagenous proteins, lipids (mainly phospholipids) and proteoglycans) of bone. ** Bio-Oss^®^ shows a Raman band also at 1075 cm^−1^ (see Supplementary Materials for details). *** Signal to noise ratio for the 1st (left) and 2nd (right) measurement.

The signal-to-noise ratio SNR at the specified wavenumber position has been given in Table 1 for both measurements separately, calculated as the ratio between the averaged Raman intensities and the root mean square (RMS) of the background noise (see Supplementary Materials for details).

**Figure 4:**
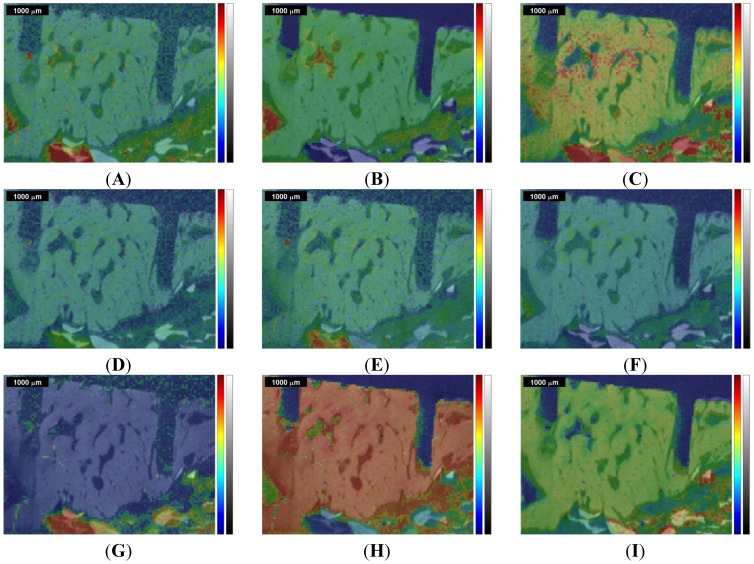
Overlap of the µ-CT image with the Raman images and correlation maps obtained within ROI 1 from the 1st measurement of the sinus grafted bone sample (compare Figure 2 and Supplementary Materials). Upper row: (**A**) Raman image centered at the CH bending peak at 1457 cm^−1^; (**B**) natural pork fat Raman correlation map; (**C**) Raman image centered at the ν_1_PO_4_^3−^ peak at 961 cm^−1^. Mid row: Raman images centered at (**D**) the B-type carbonate substitution peak at 1075 cm^−1^; (**E**) the amide I peak at 1677 cm^−1^; (**F**) the CH stretching peak at 2937 cm^−1^. Lower row: correlation of the individual spectra of the Raman map with the (**G**) Bio-Oss^®^ Raman spectrum; (**H**) natural pork bone Raman spectrum; (**I**) tricalcium phosphate Raman spectrum.

**Figure 5:**
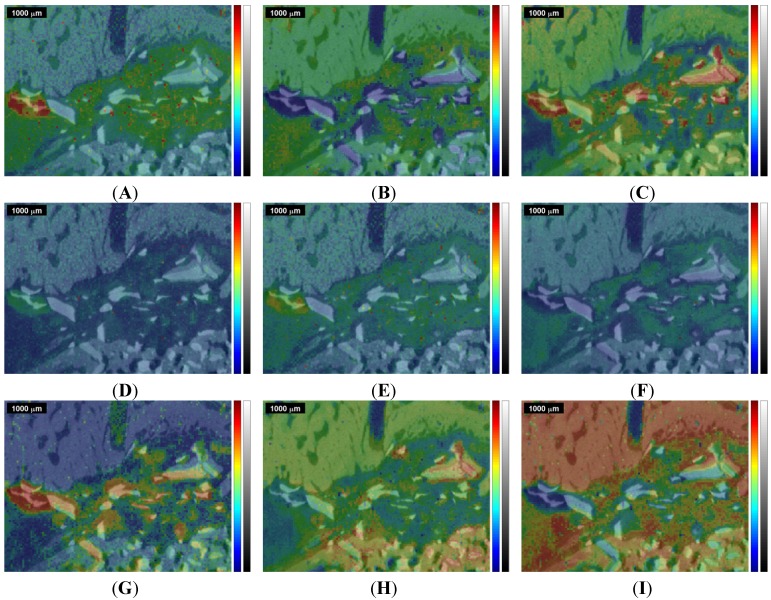
Overlap of the µ-CT image with the Raman images and correlation maps obtained within ROI 2 from the 2nd measurement of the sinus grafted bone sample (compare Figure 3 and supplementary information). Upper row: (**A**) Raman image centered at the CH bending peak at 1457 cm^−1^; (**B**) natural pork fat Raman correlation map; (**C**) Raman image centered at the ν_1_PO_4_^3−^ peak at 961 cm^−1^. Mid row: Raman images centered at (**D**) the B-type carbonate substitution peak at 1075 cm^−1^; (**E**) the amide I peak at 1677 cm^−1^; (**F**) the CH stretching peak at 2937 cm^−1^. Lower row: correlation of the individual spectra of the Raman map with the (**G**) Bio-Oss^®^ Raman spectrum; (**H**) natural pork bone Raman spectrum; (**I**) tricalcium phosphate Raman spectrum.

By means of the two Raman maps obtained in the measurement of ROI 1 and ROI 2 of the bone sample, and of Raman reference spectra (both extending through the entire spectral range detectable by the Thermo DXR Raman microscope) the Thermo Omnic 8 software allows the computation of Raman correlation maps (shown in Figure 2, Figure 3, Figure 4 and Figure 5). Since the sample originates from a previous medical study [14], the composition of the sample is well known, and therefore well suited reference substances could be chosen. The reference Raman spectra were directly obtained from Bio-Oss^®^, Bio-Gide^®^ (Geistlich Pharma AG, Wolhusen, Switzerland), tricalcium phosphate, natural untreated pork fat and natural untreated pork bone [38] (see Supplementary Materials for details). The tricalcium phosphate reference sample had a purity of 99.9%, while pork fat and bone were obtained from the local butcher and were immediately used (*i.e.*, without storage). Bio-Oss^®^ and Bio-Gide^®^ by Geistlich Pharma AG (Wolhusen, Switzerland) have been used for years in the field of bone regeneration, being probably one of the best documented bone substitute materials [39,40,41,42,43,44]. Bio-Oss^®^ refers to a deproteinized cancellous bone granulate derived from bovine bone, supporting new bone formation *inter alia* due to its osteoconductive properties [39,40]. Bio-Gide^®^ by Geistlich Pharma AG is a naturally resorbable membrane consisting of highly purified porcine type I and III collagen; it is commonly used with a bone substitute as it promotes wound healing and protects the augmented site during healing, and has been tested in several comparative studies as well [41,42,43,44].

The generation of the Raman images and the Raman correlation maps was carried out in the software solution OMNIC 8. All further statistical analysis including the image segmentation by cluster analysis and ANOVA of the image data gained by µ-CT and Raman spectroscopic imaging were performed using software routines provided by the computing environment MATLAB (Release 2011a, The Mathworks, Inc., Natick, MA, USA). The details are described further down. Note that, although the images in the present study are displayed in these pseudo RGB colors, all further calculations were carried out on the basis of gray scale values.

The Raman images and the Raman correlation maps were at first reviewed on the basis of a correlation matrix, in which the coefficients of correlation (COC) were calculated following Pearson’s ratio, defined as the ratio of the covariance of two variables (numerator) and their standard deviations (denominator) [32]. All pairs showing a COC greater than ±0.9 were discriminated, removing one element of each pair in order to avoid redundant information.

**Figure 6:**
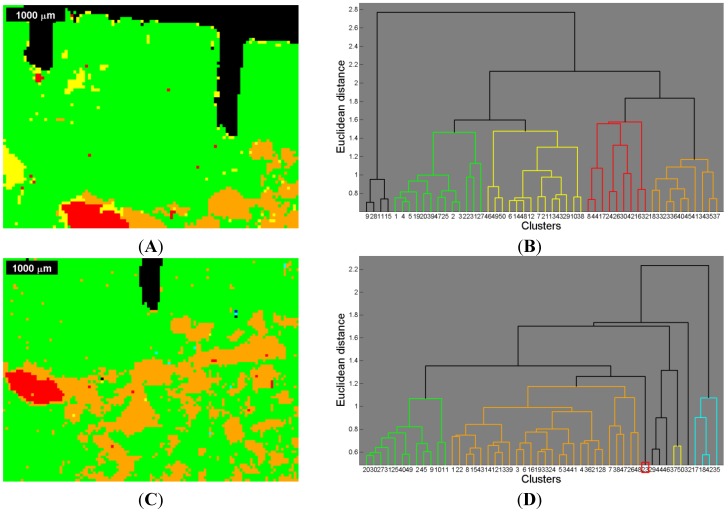
Image segmentation by cluster analysis of the Raman images and Raman correlation maps (compare Figure 2 and Figure 3), and corresponding dendrogram (**A**,**B**) for the first measurement (ROI 1) of the sinus grafted bone sample; and (**C**,**D**) for the second measurement (ROI 2) of the same sample. See also Table 3 and Table 4 for the association of a color to a given material: red—Bio-Oss^®^, orange—phosphate, green—pork bone, yellow—pork fat.

This image segmentation according to the Raman correlation maps and Raman images was realized by performing an agglomerative, hierarchical cluster analysis, which is a suitable chemometric tool to detect information parameters within a Raman based dataset [16,33,45,46]. In the present study, each image matrix representing a Raman correlation map or a Raman image, was transformed into a vector whereby all vectors subsequently formed the raw data matrix containing gray scale values in the range 0–255. The distance matrix (*i.e.*, representing the distances between each pixel) was then calculated on the basis of the raw data matrix using the Euclidean distance and an unweighted average distance between the clusters. In this manner, similar pixels (*i.e.*, pixels showing low Euclidian distances to each other) were merged in clusters until all pixels were subordinated to an overriding cluster. In order to distinguish different clusters within the overriding cluster, a threshold was set in order to avoid over- and under-segmentation; thus the number of clusters did not need to be defined previously. The results of the cluster analysis are presented in dendrograms (Figure 6B,D), in which different trees representing clusters being below the threshold are depicted in different colors. Subsequently, the results shown in the dendrograms were reconstructed into a segmented image (Figure 6A,C).

**Figure 7:**
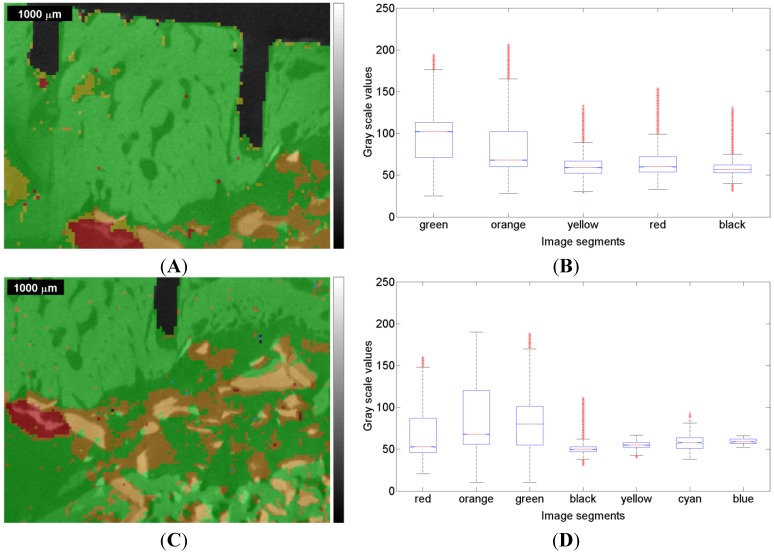
Raman based image segmentation (see Figure 6) overlapped with the µ-CT image represented by gray scale values, and box plot for the one-way ANOVA test result for the first measurement (ROI 1) of the sinus grafted bone sample (**A**,**B**); and for the second measurement (ROI 2) of the same sample (**C**,**D**). See also Table 3 and Table 4 for the association of a color to a given material: red—Bio-Oss^®^, orange—phosphate, green—pork bone, yellow—pork fat.

The image showing the segmentation based on the Raman images and the Raman correlation maps was overlapped with the µ-CT image (Figure 7A,C) in order determine the relationship of the gray scale values (µ-CT) with the Raman based segmentation image by means of an one-way analysis of variance (one-way ANOVA), (Figure 7B,D) [45].

In addition, Table 3 and Table 4 (Section 3) show the explanatory amount of the correlation maps. The coefficient of determination (COD), which is defined as the square of the COC, was calculated by the Raman software OMNIC 8, and is linearly scaled with the intensities given in color in the correlation maps. Therefore, a COD could be calculated for each colored segment within the Raman based segmented image.

## 3. Results and Discussion

The Raman images and Raman correlation maps with redundant information according to the correlation matrix were removed from the data sets for the first and second measurement and are listed separately in Table 2.

**Table 2:** Raman images and Raman correlation maps removed from the data set.

Raman Peak	Peak Position [cm^−1^]	Raman Peak	Peak Position [cm^−1^]
*1st Measurement*		*2nd Measurement*	
Bio-Gide ^®^	–	Bio-Gide ^®^	–
Amide III	1256	Amide III	1256
ν_4_PO_4_^3−^	589	ν_4_PO_4_^3−^	589
ν_2_PO_4_^3−^	438	–	–

Figure 2 shows the results of the first measurement (ROI 1) and Figure 3 shows the results for the second measurement (ROI 2). In the upper row, Figure 2A displays the microscopic image of the sinus grafted bone sample, with the two kerfs as orientation marks, and Figure 2B the corresponding µ-CT image. The Raman image shown in Figure 2C displays the intensities (red being high, blue being low) of the Raman peak centered at 961 cm^−1^, attributed to the ν_1_ stretching vibration of PO_4_^3−^ [21,23]. In the mid row, three Raman images are shown: Figure 2D displays the intensities at 1075 cm^−1^ (B-type carbonate substitution), Figure 2E those at 1677 cm^−1^ (amide I), and Figure 2F those at 2937 cm^−1^ (CH-stretching mode). In the lower row, three Raman correlation maps are shown, red meaning high, and blue meaning low correlation of the individual spectra of the original Raman map with the chosen reference spectrum. Figure 2G displays the correlation with the Bio-Oss^®^ Raman spectrum, Figure 2H that with the pork bone Raman spectrum, and Figure 2I that with the tricalcium phosphate Raman spectrum. Additional Raman images and correlation maps are shown in the Supplementary Materials. Figure 4 shows a selection of Raman images and correlation maps overlapped with the µ-CT image of ROI 1 (Figure 2B) by transparency mapping methods in MATLAB, the digital resolution of the µ-CT image being adapted to that of the Raman images by using image-processing tools in MATLAB.

A distinct assignment of tissues or materials (e.g., bone, Bio Oss^®^, fat, organic matrix, phosphate and carbonate mineral) just by comparing the overlapped images with each other turns out to be challenging, apart from the fact that the correlations with the Raman spectrum of Bio Oss^®^ (Figure 2G and Figure 3G) and of natural pork bone (Figure 2H and Figure 4H) behave rather disjunctively to each other.

The Bio-Oss^®^ correlation map (Figure 2G and Figure 4G) shows, in its bottom left-hand and right-hand corner, areas of high correlation that largely match, within the Raman image centered at the ν_1_PO_4_^3−^ peak at 961 cm^−1^ (Figure 2C and Figure 4C), the corresponding areas with high ν_1_PO_4_^3−^ Raman intensities, these indicating higher concentration of phosphate mineral, *i.e.*, above the level naturally present in bone.

Moreover, the Raman images Figure 2D, centered at 1075 cm^−1^ (B-type carbonate substitution), agree pretty well with the Bio-Oss^®^ correlation map shown in Figure 2G; both can be well discriminated when comparing them with the other Raman images and Raman correlation maps of Figure 2. Since carbonated hydroxyapatite is also used in bone graft substitutes due to its chemical similarity to bone mineral [47], and Bio-Oss^®^ is reported to contain carbonate apatite [48], areas in a Raman image with a high intensity at 1075 cm^−1^ can thus be considered as an indicator for Bio-Oss^®^ too.

The tricalcium phosphate correlation map itself (Figure 2I and Figure 4I) cannot be considered as indicator for bone (compare Figure 2H and Figure 4H), since areas of high correlation with tricalcium phosphate (in red in Figure 2I and Figure 4I) behave rather disjunctively to those areas of high correlation with pork bone (in red in Figure 2H and Figure 4H). On closer inspection, the tricalcium phosphate correlation map (Figure 2I and Figure 4I) also describes any deviations due to presence of organic moieties of bone (collagen, non-collagenous proteins, lipids and proteoglycans), see also the Raman images and Raman correlation maps in the Supplementary Materials. Moreover, the high correlation areas within the tricalcium phosphate correlation map (in red in Figure 2I and Figure 4I) do not show a full coincidence with the corresponding areas with high ν_1_PO_4_^3−^ Raman intensities (red areas in Figure 2C and Figure 4C) and with high correlation areas in the Bio-Oss^®^ correlation map (in red in Figure 2G and Figure 4G), thus tricalcium phosphate does not appear to be unambiguously assignable (see also Figure 6 in the discussion section).

A second Raman spectroscopic measurement of the same sample, with ROI 2 partially overlapping ROI 1 (see Figure 1), was performed, primarily to see if equivalent conclusions to the first measurement could be obtained. The results for ROI 2 are given in Figure 3 and Figure 5 in the same manner as for ROI 1 in Figure 2 and Figure 4, the kerf in ROI 2 being the same one as the long kerf in the upper-right corner of ROI 1 (see Figure 1). The comparison of Figure 3 with Figure 2 and of Figure 5 with Figure 4 confirms the equivalent information content within the overlapping ROIs. Here, the same conclusions can be derived as in the first measurement.

The area that highly correlates with Bio Oss^®^ in the mid-left of Figure 2G, Figure 3G, Figure 4G and Figure 5G also shows pronounced intensity at 1258 cm^−1^ (amide III) and at 1457 cm^−1^ (CH bending mode), as shown in the figures in the Supplementary Materials and in Figure 4A and Figure 5A, and also at 1677 cm^−1^ (amide I), as shown in Figure 2E, Figure 3E, Figure 4E and Figure 5E. This is due to the presence of luminescence in the spectral region between 1200 cm^−1^ and 2000 cm^−1^ produced by the inorganic components of Bio Oss^®^ [49].

These facts highlight that an ordinary interpretation following Table 1 is not straightforward, and emphasize the need to implement statistical calculation methods in order to achieve one image, containing the complete chemical information gained by Raman spectroscopic imaging, that can then be compared with the µ-CT image. Furthermore, in Figure 4 and Figure 5 one can recognize that all differently colored Raman regions can cover both bright gray scale values (high X-ray absorption, *i.e.*, high density) as well as dark ones (low X-ray absorption, *i.e.*, low density), thus an assignment of different density values to specific vibrational Raman modes is not useful at present.

**Figure 8:**
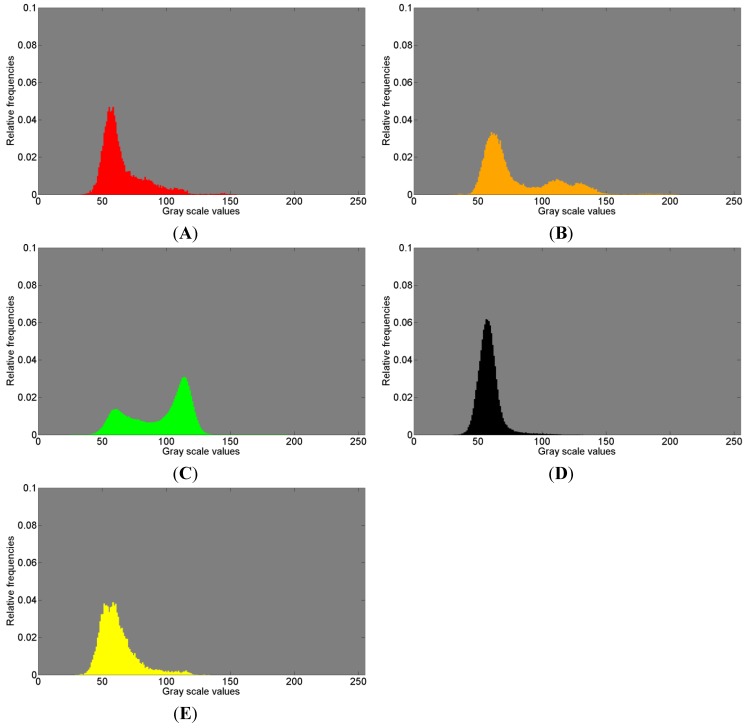
Histogram of gray scale values for (**A**) red; (**B**) orange; (**C**) green; (**D**) black; (**E**) yellow, shown in Figure 6A and in Figure 7A, associated with the first measurement (ROI 1) of the sinus grafted bone sample. See also Table 3 for the association of a color to a given material: red—Bio-Oss^®^, orange—phosphate, green—pork bone, yellow—pork fat.

**Figure 9:**
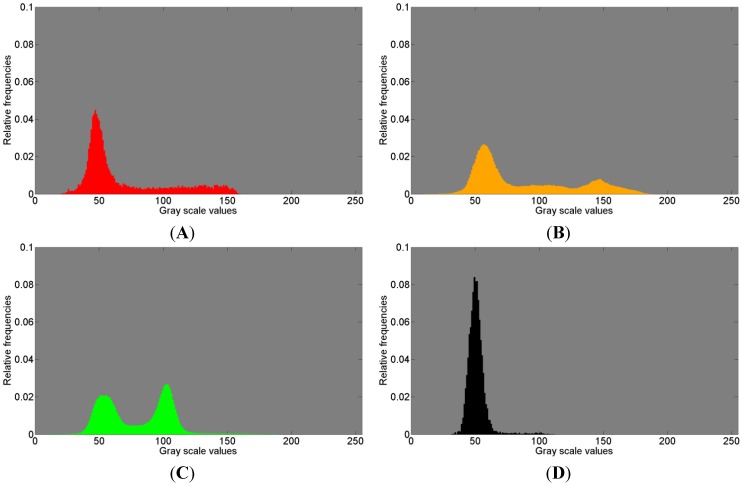
Histogram of gray scale values for (**A**) red; (**B**) orange; (**C**) green; (**D**) black, shown in Figure 6C and in Figure 7C, associated with the second measurement (ROI 2) of the sinus grafted bone sample. See also Table 4 for the association of a color to a given material: red—Bio-Oss^®^, orange—phosphate, green—pork bone.

In order to create a segmented image based on the Raman data set containing the chemical information of all Raman images and Raman correlation maps (see Figure 6), an agglomerative, hierarchical cluster analysis was performed. This segmented image was used afterwards for comparative purposes (by means of a one-way ANOVA) regarding the gray scale values of the µ-CT image (see Figure 7, Figure 8 and Figure 9). Note that, although the Raman images are displayed in pseudo RGB colors the calculations were carried out on the basis of gray scale images with values ranging from 0 to 255.

In the agglomerative, hierarchical cluster analysis a threshold in distance (*i.e.*, ordinate) had to be set in order to obtain distinguishable cluster trees within the overriding cluster tree. The threshold was manually optimized in order to avoid over- and under-segmentation respectively, and set at 57% of the total (Euclidean) distance for the first measurement, and at 55% of the total distance for the second measurement.

Due to the thresholds, different segmentation areas could be determined resulting in a segmented image (see Figure 6A,C) based on the Raman images and Raman correlation maps shown in Figure 2 and Figure 3 and in the Supplementary Materials. The different trees within the dendrograms of Figure 6 B and Figure 6D are color-coordinated to the segmentation in the image. Both dendrogram plots show 50 nodes at the bottom (*i.e.*, abscissa), cutting lower parts of the respective tree and thus enabling a better presentation.

For the first measurement, when comparing the Raman based image segmentation (Figure 6A) with the respective Raman images and Raman correlation maps (Figure 2 and Figure 4), red indicates the presence of Bio-Oss^®^, green the presence of pork bone, and yellow the presence of lipids. In the orange colored areas, the presence of both the phosphate band at 961 cm^−1^ (Figure 2C and Figure 4C), Bio-Oss^®^ (Figure 2G and Figure 4G), and tricalcium phosphate (Figure 2I and Figure 4I) were rather high, whereas the presence of pork bone (Figure 2H and Figure 4H) was rather low, suggesting that these are areas in which Bio-Oss^®^ was resorbed.

The same approach used in the first measurement was applied to the second measurement using the same methods. Note that the red area in Figure 6C cannot be spotted in the dendrogram of Figure 6D since the appropriate tree (*i.e.*, at the node 23 marked in red) is situated below the horizontal axis (*i.e.*, abscissa). As mentioned before, lower parts within the dendrogram were clipped, affecting the red colored area precisely there.

The presence of faulty measurements (*i.e.*, poor SNR), which are arbitrarily distributed in the image and are most noticeable in Figure 2D,E and Figure 3D,E, were discriminated by thresholding the total (Euclidean) distance and assigning different trees to these faulty measurements, as far as this was possible. To a limited degree, some faulty measurements still proved to be part of a reasonable segmentation color.

The segmentation areas on the basis of the Raman images and correlation maps were overlaid with the µ-CT image (Figure 7A,C). The corresponding µ-CT gray scale values underwent a one-way ANOVA [50], the results in the box plot (Figure 7B,D) showing how the gray scale values on the ordinate are linked up to particular segmentation areas identified by appropriate colors on the abscissa (compare Figure 6). On each box, the central mark is the median, the edges of the box are the 25th and 75th percentiles, the whiskers extend to the most extreme data points not considered outliers, and outliers are individually plotted.

In the first measurement, one can observe a notably high variance of the gray scale values within each color. The medians related to a particular color are all in the range of approximately 60–110, which is nearly 20% of the total range (*i.e.*, 0–255).

In the second measurement, the gray scale values show a high variance as well, the medians being in the 50–80 range, which is even narrower than in the first measurement. This might be due to the fact that the bone sample had already dried up at the beginning of the second measurement, but in the end the ranges of gray scale values are narrow in both measurements. The high variance with respect to both measurements leads to the question of how that variance can be explained.

Figure 8, referring to the first measurements, and Figure 9, referring to the second measurement, show the normalized histograms of the gray scale values for the segmentation areas in images Figure 6A,C and Figure 7A,C with their respective colors. The gray scale values are displayed on the abscissa and the relative counts on the ordinate.

The red colored gray value distributions in Figure 8A and Figure 9A clearly show one peak and a large amount of widely spread gray scale values that possibly represent a second distribution, thus presumably indicating the presence of another tissue.

The orange histograms in Figure 8B and Figure 9B clearly show two distributions and possibly a third distribution in between. Three different distributions possibly indicating three materials different in density suggest some resorption activities of Bio-Oss^®^.

In Figure 8C, the green histogram covers a large amount of scattered gray scale values and reveals a bimodal distribution. This clearly indicates that the variance appears due to the fact that at least two distributions are overlapped resulting in an accumulation around the gray scale values of 60 and 115, respectively. This means that within the Raman based image segmentation, one area consists of darker gray scale values (around 60) and a different area comprises brighter gray scale values (around 115), each likely representing a different material. This should be considered when watching the box plots related to the ANOVA test results (in this case Figure 7B). The green colored segment part related to the second measurement reveals a similar histogram (Figure 9C, compare Figure 7D) with at least two distributions clearly overlapping. In summary, the green histograms suggest the presence of more than one material.

The black histograms (Figure 8D and Figure 9D, compare Figure 7B,D) show a single distribution with a small amount of widely scattered gray scale values.

The yellow histogram (Figure 8E, compare Figure 7B) shows two peaks very close to each other. This leads to the conclusion that two distributions overlap to a considerable extent.

In summary, close similarities within each color of the histograms can be observed with respect to the 1st and 2nd measurements. The histograms in Figure 8 and in Figure 9 clearly indicate, in addition to the visual perception (Figure 7A,C), that the µ-CT information (*i.e.*, density) does not necessarily fit the Raman spectra in such a manner that a certain assignment of tissues can be made. In order to give a potential answer as to what kind of tissue the Raman based image segmentation (*i.e.*, cluster analysis) represents, the Raman correlation maps were scaled with the appropriate coefficients of determination (COD) obtained with the software OMNIC 8. The results are given in Table 3 and Table 4, respectively, in which the highest COD value in a row associates a color to a given material.

Following Table 3 (related to the first measurement), Bio-Oss^®^ can be explained most suitably with the color red (0.74), pork bone with green (0.78), and finally pork fat being represented by yellow (0.51). Tricalcium phosphate cannot be clearly allocated to any particular color since the CODs are all greater than 0.66 (except black being the saw kerf). This reference measurement was not found to be statistically significant according to the *t*-test in the multiple linear model [37].

**Table 3:** Coefficient of determination (COD) related to the image segmentation based on the Raman correlation maps of the 1st measurement (compare Figure 6A,B). The highest COD value in a row associates a color to a given material.

Material	Red	Orange	Green	Black	Yellow
Bio-Oss^®^	0.74	0.57	0.16	0.19	0.27
Tricalcium phosphate	0.68	0.82	0.77	0.60	0.66
Pork bone	0.17	0.39	0.78	0.14	0.53
Pork fat	0.14	0.21	0.43	0.13	0.51

Table 4 (related to the second measurement) again reveals that red indicates Bio-Oss^®^ (0.80). The color green represents primarily pork bone; pork fat shows low CODs, with 0.45 as the highest associated with green too. Similarly in Table 3, tricalcium phosphate is highly represented in every color, however, the same conclusion can be drawn in these terms as in the first measurement.

**Table 4:** Coefficient of determination (COD) related to image segmentation based on the Raman correlation maps of the second measurement (compare Figure 6C,D). The highest COD value in a row associates a color to a given material.

Material	Red	Orange	Green	Black
Bio-Oss^®^	**0.80**	0.58	0.24	0.34
Tricalcium phosphate	0.69	*0.79*	0.77	0.64
Pork bone	0.14	0.39	**0.71**	0.17
Pork fat	0.16	0.25	0.45	0.18

However, taking into account the observations made in terms of the tricalcium phosphate correlation map (Figure 2I and Figure 4I, respectively, Figure 3I and Figure 5I) and the ν_1_PO_4_^3−^ Raman image at 961 cm^−1^ (Figure 2C and Figure 4C, respectively, Figure 3C and Figure 5C) and considering the results of the cluster analysis (Figure 6A,C), it can be seen that the orange segmentation area matches the high intensities in the tricalcium phosphate correlation map and the ν_1_PO_4_^3−^ Raman image at 961 cm^−1^ to a very great extent. Furthermore, the overlap with the µ-CT image (Figure 7A,C) reveals high density values in this area as well, thus the orange segmentation area appears to indicate some kind of mineralization zone. Thus the reference spectrum of tricalcium phosphate (see Supplementary Materials) seems to be suitable in order to determine regions likely representing mineralization areas. This assumption is first supported by the visual observation that high correlation values in the correlation maps of tricalcium phosphate (Figure 2I and Figure 3I) partially match high values of the Bio-Oss^®^ and the pork bone correlation map (compare Figure 2G,H, and Figure 3G,H). Second, the so called mineralization area was determined as a separate area according to the hierarchical agglomerative cluster analysis (orange segments in Figure 6). Third, following a closer inspection, the high correlation values in the tricalcium phosphate correlation maps (Figure 4I and Figure 5I) are fairly located along the edges of high density values in the µ-CT image, likely representing bone. This emphasizes the fact that tricalcium phosphate seems to be an indicator for a mineralization area. Consequently, the high density areas within the orange area seem to be newly formed bone. Although it is right, that according to Table 3 tricalcium phosphate seems to be represented in every area, the highest COD can be located for the orange area.

In summary, Bio-Oss^®^ and pork bone can be assigned with a fair degree of certainty. Pork fat could easily be determined in the first measurement, being represented with yellow (compare also the Raman correlation maps in the Supplementary Materials). The second measurement evidently did not comprise some lipid areas as in the first measurement.

Tricalcium phosphate seems to be part of all other materials since the COD is relatively high in all materials for both measurements, however, the COD takes on the largest value for the so called mineralization area. Considering the fact that bone consists of a large number of different calcium phosphates due to ionic substitutions, an ordinary tricalcium phosphate powder as a reference is probably insufficient in order to make more precise statements here. Although bone apatite can be differentiated from synthetic hydroxyapatite (OHAp) by Raman spectroscopy [47], a sophisticated analysis of the occurrence of different apatitic phosphates in the sample was not included in the present study, thus, this should be part of further studies.

## 4. Conclusions

This study shows how different Raman images and Raman correlation maps can successfully be used to generate a segmented image comprising the biochemical information by means of hierarchical cluster analysis. Thereby, the Raman correlation maps of the known compounds (*i.e.*, Bio-Oss^®^, Bio-Gide^®^, natural pork bone and natural pork fat) resulted in valuable information since the bone graft substitute Bio-Oss^®^ and pork bone could clearly be discriminated by Raman spectroscopy. Without these additional correlation maps, the segmentation result by cluster analysis would not be as detailed as given in Figure 6A,C; therefore, general knowledge of the composition of the object of interest is a prerequisite for reliable segmentation results based on Raman spectra. Although the explanatory amount of the Raman correlation map of tricalcium phosphate seems to be rather low according to Table 3 and Table 4, this correlation map turned out to be useful to assess the orange area within the segmentation result as mineralization area. Therefore, mineralization areas are assumed to be detectable by this method, and thus a distinction between original bone, autogenous bone and new bone formation being in process at the time of sampling, can be made by Raman spectroscopy.

The Raman based image segmentation is partially in agreement with the density information gained by µ-CT. Nevertheless, all segments found in the Raman based segmentation image (Figure 6) cover a broad range of gray scale values in the µ-CT image and, therefore, restrict a clear assignment of tissues or materials solely by means of µ-CT. This conclusion is emphasized by the results according to the one-way ANOVA (Figure 7) showing that reliable conclusions on biochemical compositions cannot be made exclusively on the bases of density information yet, since no typical gray scale values from the µ-CT image could be attributed to areas found in the Raman image (see Figure 7).

Thus, the hypothesis that a methodological differentiation between autogenous bone and newly formed bone by means of Raman spectroscopy is possible can be confirmed. A methodological distinction based exclusively on µ-CT could not be made, since no typical gray scale values corresponding to the chemical information could be found.

The histograms in Figure 8 and Figure 9 suggest that a more sophisticated differentiation on the basis of µ-CT is likely possible within a chemically determined area. Beyond that, different additional imaging techniques such as magnetic resonance imaging (MRI) or infrared spectroscopic imaging (IR) could contribute to improvements in this field, however, this was not further investigated in the present study and should be part of future research efforts.

The Raman images showing solely medium to low intensities (Figure 2D,E and Figure 3D,E) were enhanced by squeezing the range of intensity in order to enhance the actual image. This should generally be discussed to what extent low intensities maps are allowed to be processed by contrast enhancement (Table 1), since contrast enhancement possibly influences the segmentation results and thus constrains comparability of different studies in this field. However, this was not further investigated within the present study.

Conversely, however, neither µ-CT nor Raman spectroscopy is the holder of the truth, instead these two different imaging techniques provide information that are complementary and mutually supportive. This consideration should be taken into account for medical diagnostic routines as well as for studies focusing on the characterization of biomaterials based on imaging techniques. Up to now, a reliable distinction between original bone, newly formed bone and mineralization areas requires chemical information.

## Supplementary Materials

Supplementary materials can be accessed at: http://www.mdpi.com/1996-1944/8/7/3831/s1.
