# Supplementary material for: A Bone Sample Containing a Bone Graft Substitute Analyzed by Correlating Density Information Obtained by X-ray Micro Tomography with Compositional Information Obtained by Raman Microscopy
Source: Materials (Basel). 2015 Jun 25;8(7):3831–53. doi: 10.3390/ma8073831 (PMC5455664; doi:10.3390/ma8073831)
Supplement: Supplementary file 1 [file materials-08-03831-s002.pdf]

Supplementary Materials

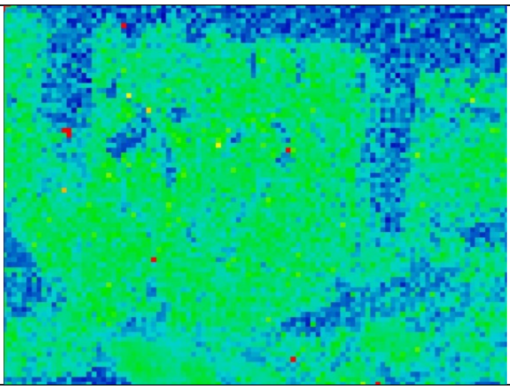	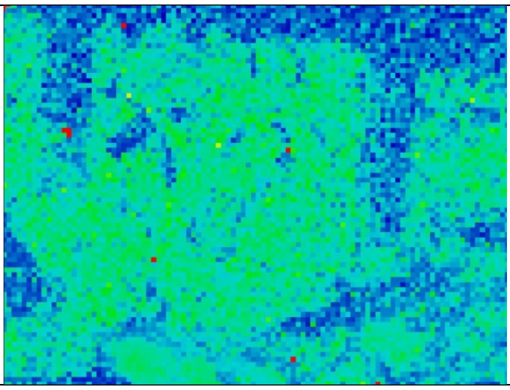
Phosphate bending mode$\nu_2PO_4^{3-}$ (438 cm^{-1})	Phosphate bending mode$\nu_4PO_4^{3-}$ (589 cm^{-1})
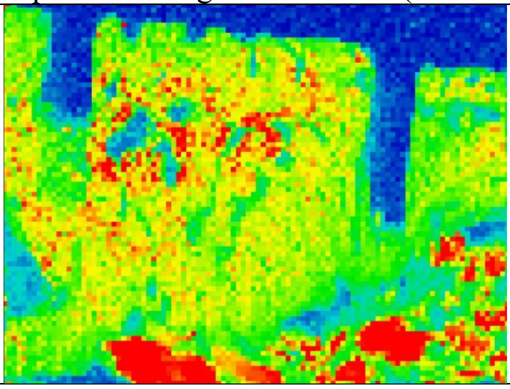	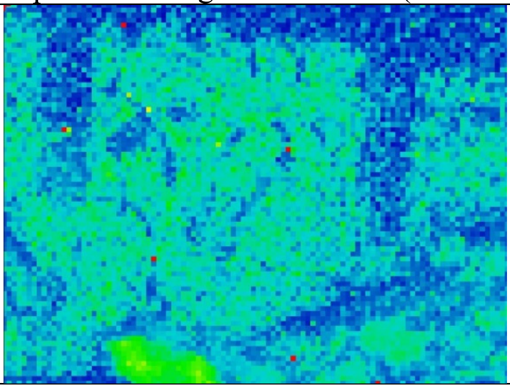
Phosphate stretching mode$\nu_1PO_4^{3-}$ (961 cm^{-1})	Type B carbonate substitution (1075 cm^{-1})
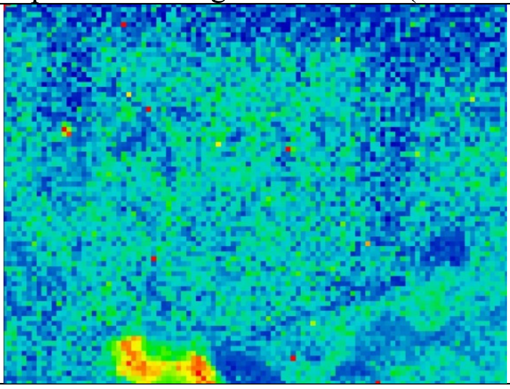	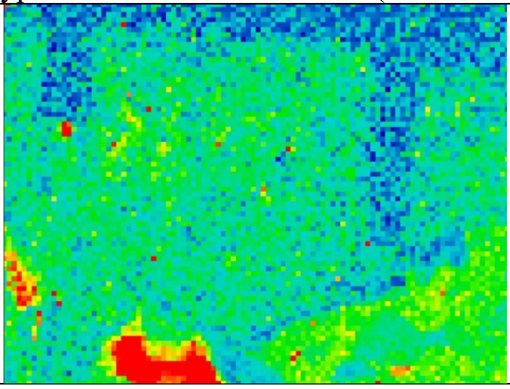
Amide III stretching mode (1258 cm^{-1})	CH bending mode (1457 cm^{-1})
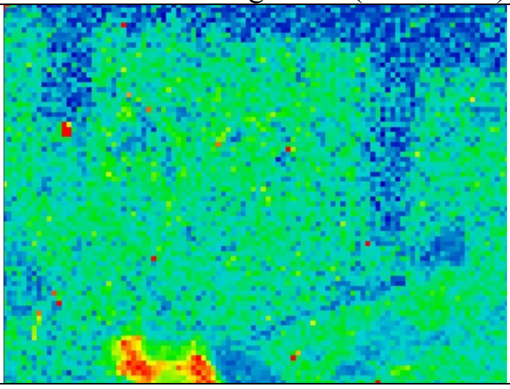	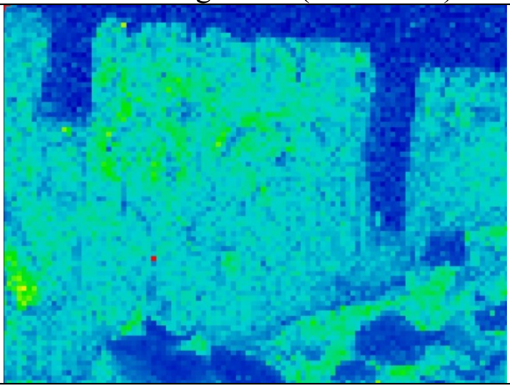
Amide I stretching mode (1676 cm^{-1})	CH stretching mode (2938 cm^{-1})

Figure S1. 1st measurement—Raman images generated by focusing on Raman bands according to Table 1. The images show the intensity distribution at a given wavenumber position [29,31].

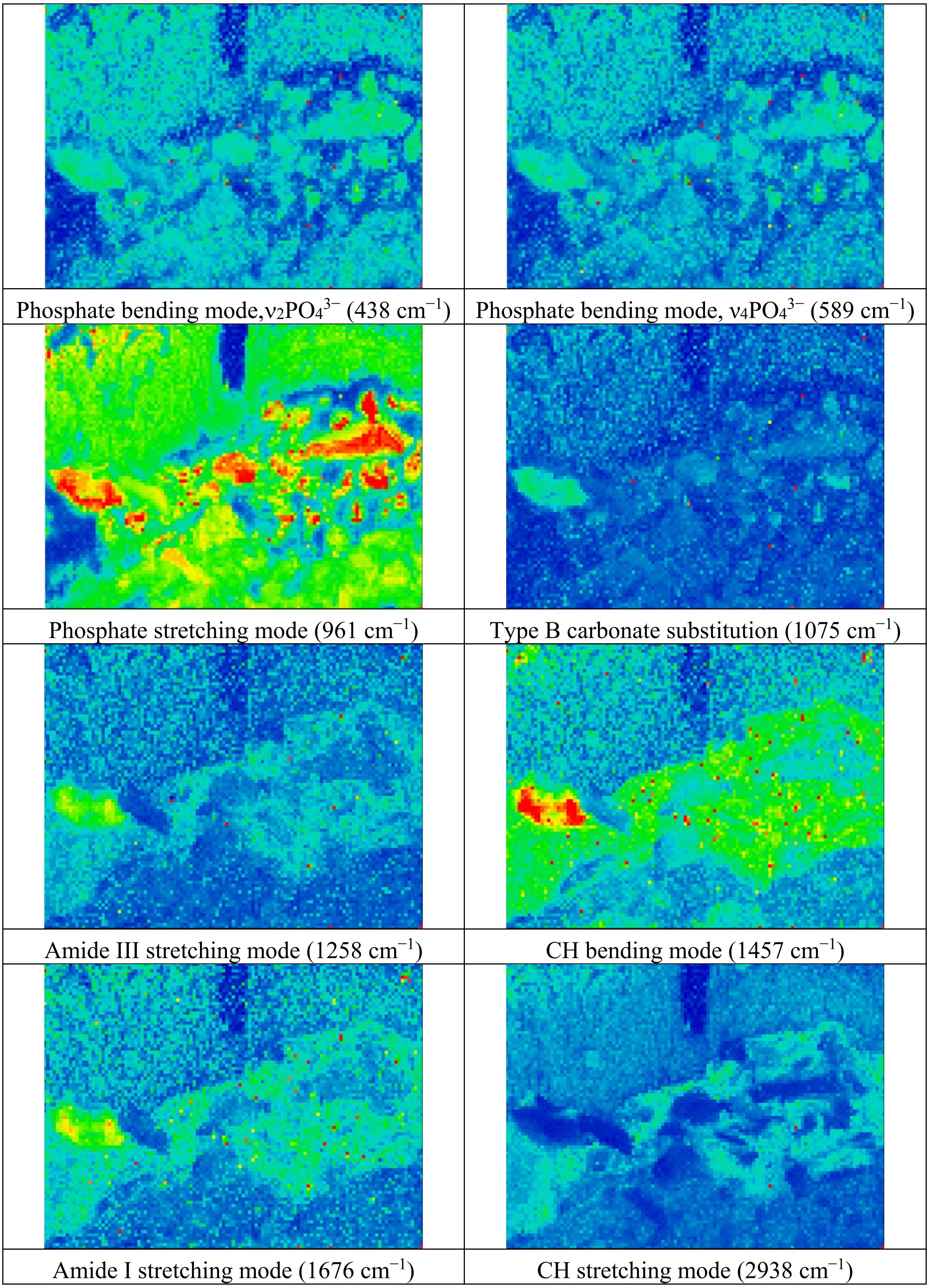

Figure S2. 2nd measurement—Raman images generated by focusing on Raman bands according to Table 1. The images show the intensity distribution at a given wavenumber position [29,31].

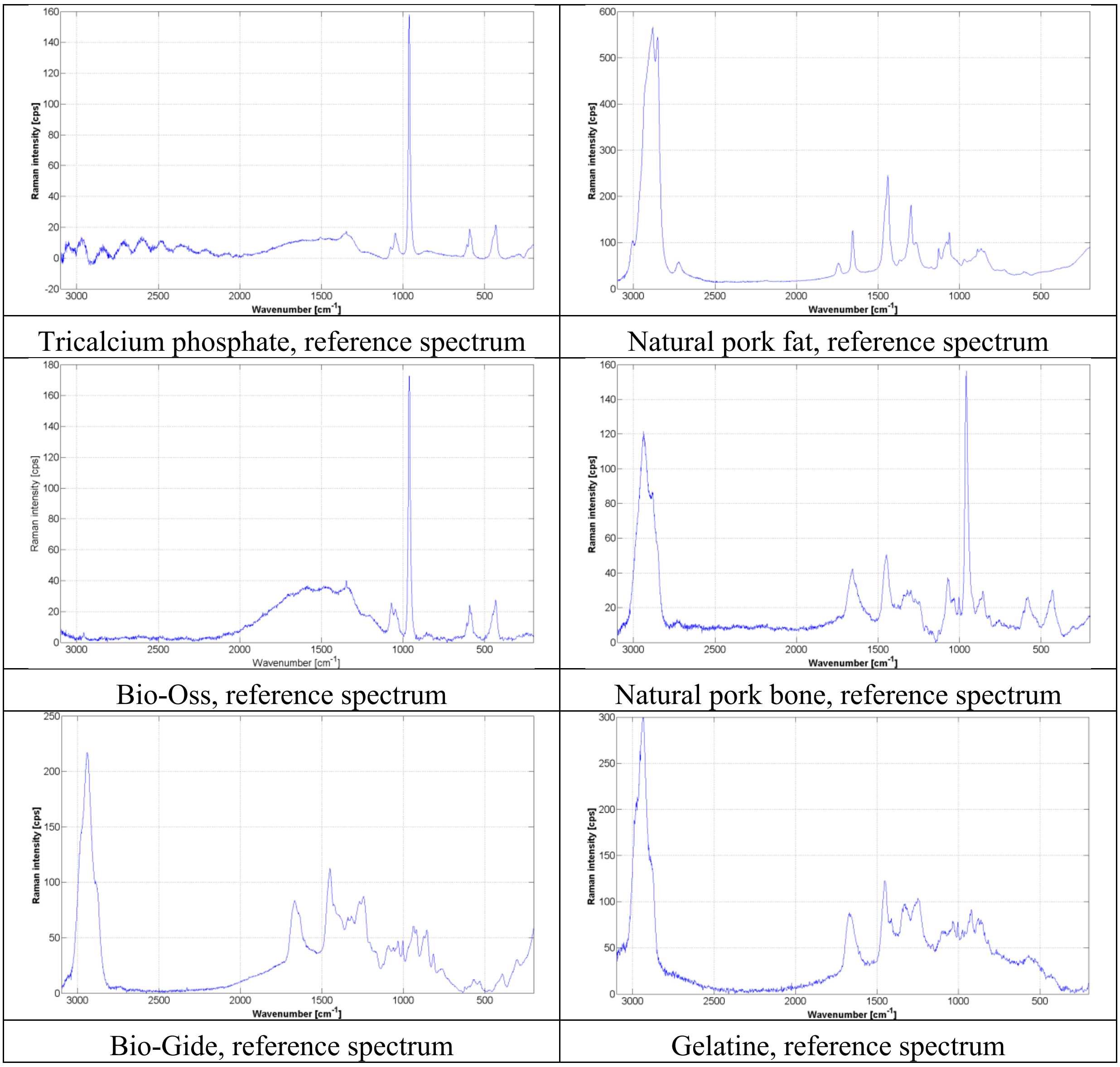

Tricalcium phosphate, reference spectrum

Natural pork fat, reference spectrum

Bio-Oss, reference spectrum

Natural pork bone, reference spectrum

Bio-Gide, reference spectrum

Gelatine, reference spectrum

Figure S3. Reference spectra used to generate Raman correlation maps.

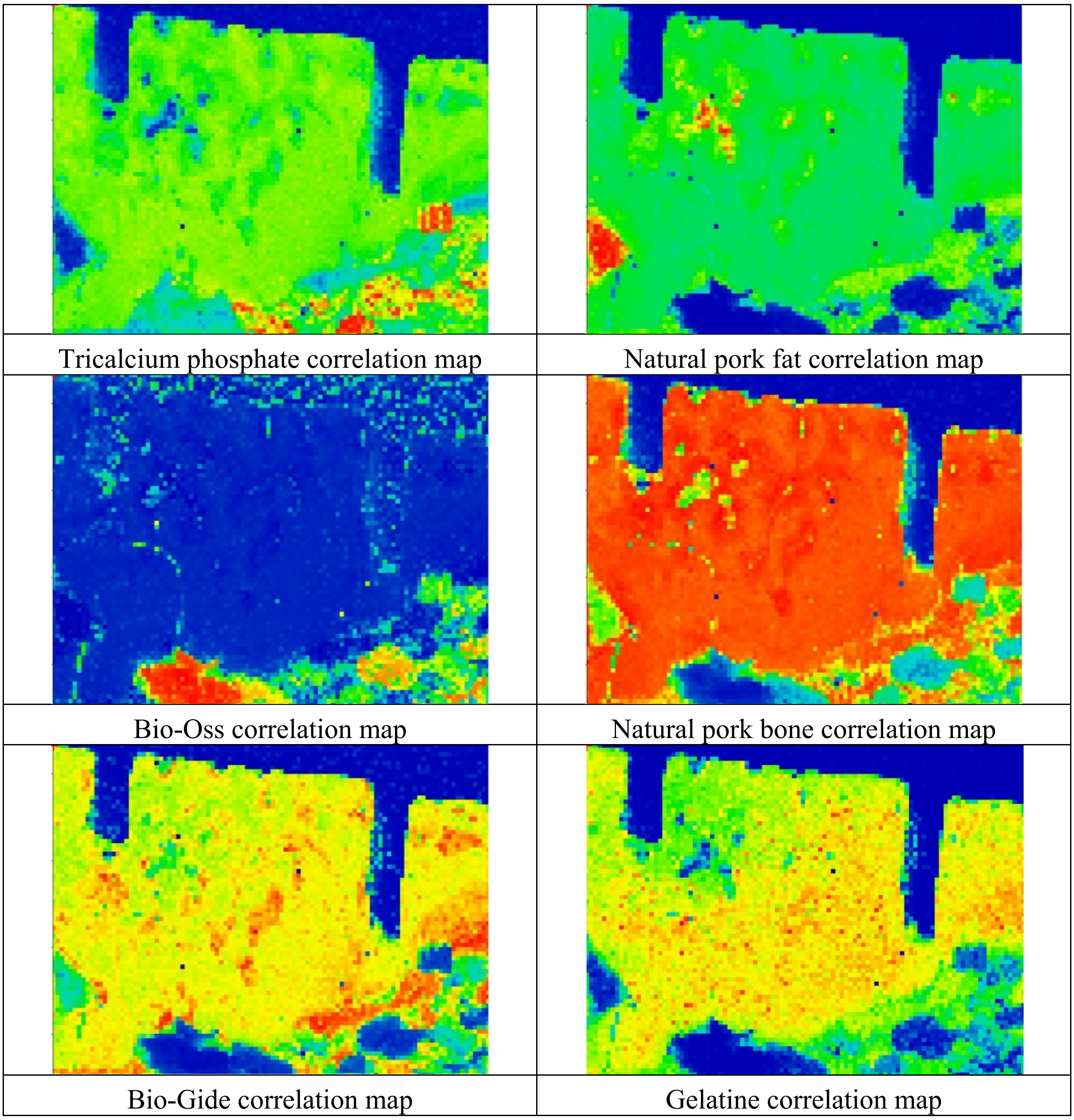

Figure S4. 1st measurement—Raman correlation maps generated by correlating the reference spectra with the basic measurement.

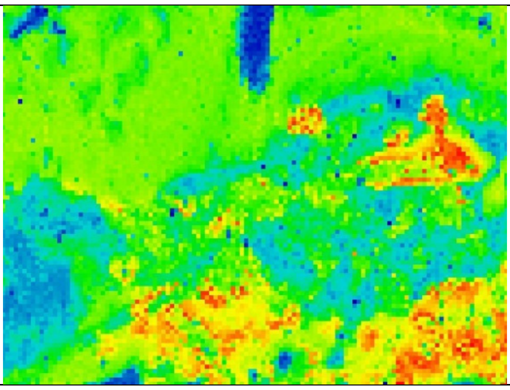	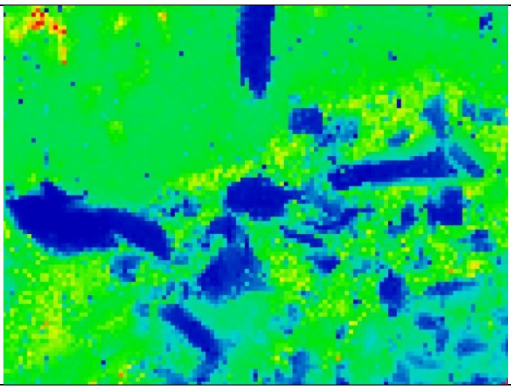
Tricalcium phosphate correlation map	Natural pork fat correlation map
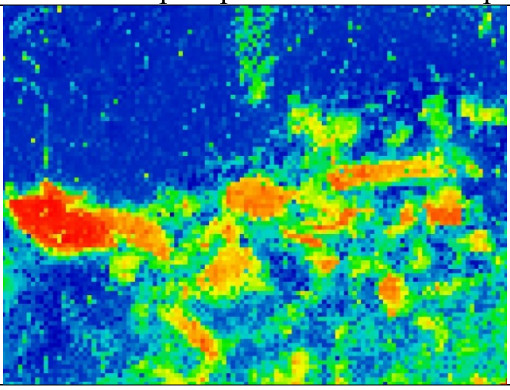	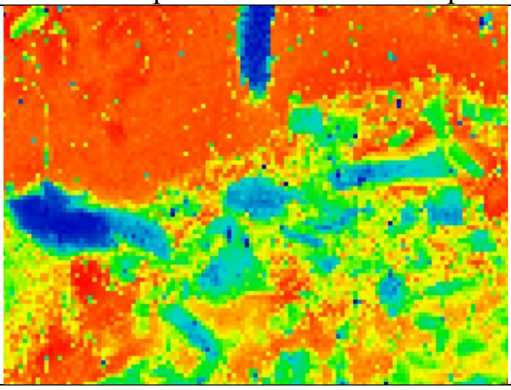
Bio-Oss correlation map	Natural pork bone correlation map
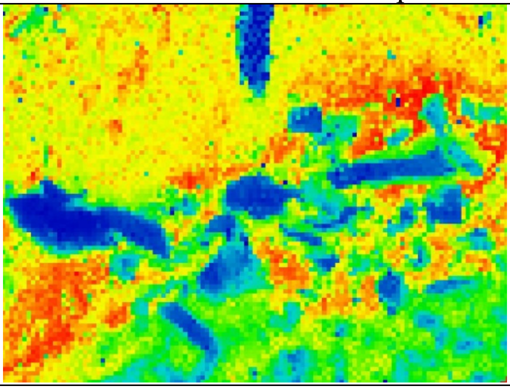	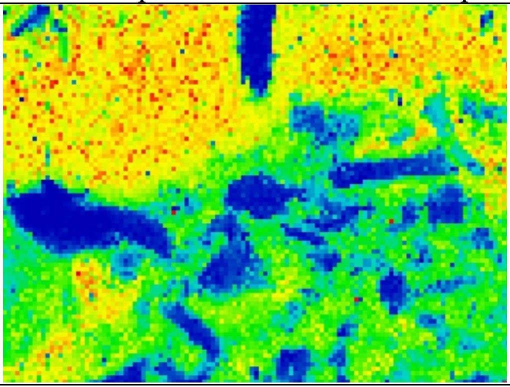
Bio-Gide correlation map	Gelatine correlation map

Figure S5. 2nd measurement—Raman correlation maps generated by correlating the reference spectra with the basic measurement.
